# Supplementary material for: Optimal Sequence and Second-Line Systemic Treatment of Patients with RAS Wild-Type Metastatic Colorectal Cancer: A Meta-Analysis
Source: J Clin Med. 2021 Nov 4;10(21):5166. doi: 10.3390/jcm10215166 (PMC8584361; doi:10.3390/jcm10215166)
Supplement: Supplementary file 1 [file jcm-10-05166-s001.zip › Supplementary_tables.pdf]

Supplementary Table S1. Keywords used for search strategies

Database	Key words
Pubmed	(((((sequence) OR third) OR second)) AND (((("metastasis"[All Fields] OR "metastases"[All Fields] OR "metastatic"[All Fields]) AND ("colorectal neoplasms"[MeSH Terms] OR "colorectal neoplasms"[All Fields] OR "colorectal cancer"[All Fields] OR "colonic neoplasms"[MeSH Terms] OR "colonic neoplasms"[All Fields] OR "colon cancer"[All Fields] OR "colon tumor"[All Fields] OR "Colorectal Carcinoma"[All Fields] OR "sigmoid cancer"[All Fields] OR "sigmoid neoplasms"[MeSH Terms] OR "sigmoid neoplasms"[All Fields])) AND (("panitumumab"[MeSH Terms] OR "panitumumab"[All Fields]) OR ("cetuximab"[MeSH Terms] OR "cetuximab"[All Fields]) OR ("bevacizumab"[MeSH Terms] OR "bevacizumab"[All Fields])))))
Cochrane library	(Panitumumab or Cetuximab or Bevacizumab) AND (Secondary or metastatic) and (''colorectal neoplasms'' or ''colorectal cancer'' or ''sigmoid neoplasms'') AND (sequence OR third OR second) and Trials
Embase	('metastatic colorectal carcinoma'/exp OR 'metastatic colorectal carcinoma' OR 'metastatic colorectal cancer'/exp OR 'metastatic colorectal cancer' OR 'colorectal tumor'/exp OR 'colorectal tumor' OR 'colon tumor'/exp OR 'colon tumor') AND ('bevacizumab' OR 'bevacizumab'/exp OR bevacizumab OR 'cetuximab' OR 'cetuximab'/exp OR cetuximab OR 'panitumumab' OR 'panitumumab'/exp OR panitumumab) AND (sequence OR third OR second) AND [controlled clinical trial]/lim

Supplementary Table S2. Characteristics and summary results of the included studies assessing efficacy in *KRAS* wild-type metastatic colorectal cancer patients.

Study	Country	Study design	Study period	ECOG status	Patient number	First-line therapy regimen		Second-line therapy regimen		Third-line therapy regimen		PFS, HR (95%CI)	OSHR (95%CI)	ORR (n/N)	
						Intervention	Comparison	I	Comparison	I	Comparison				
Bennouna et al. 2019 [1] (PRODIGE18)	France	RCT	Dec 2010 - May 2015	0-1	I: 65 C: 67	FOLFOX+ B or FOLFIRI+ B	FOLFOX + B or FOLFIRI + B	FOLFIRI + B or FOLFOX + B	FOLFIRI + Cet or FOLFOX + Cet	N/A	N/A	0.71 (0.5-1.02)	0.69 (0.46-1.04)	16/65	20/67
Buchler et al. 2019[2]	Brno	Prospective cohort, non-interventional post-registration database		0-2	I:309 C:181	FOLFOX or FOLFIR + B	FOLFOX or FOLFIR + Cet or P	FOLFOX or FOLFIR + Cet or P	FOLFOX or FOLFIR + B	N/A	N/A	0.68 (0.53–0.87)	1.12 (0.77–1.65)	N/A	N/A
Cascinu et al. 2017 [3] (COMETS)	Italy	Multicenter, RCT	Sep 2009-Apr 2015	0-1	I: 55 C: 53 L: 36/38 R:16/15	FOLFIRI+ B	FOLFIRI+ B	FOLFOX-4	irinotecan/Cet	Irinotecan /Cet	FOLFOX-4	1.04 (0.69-1.56) L: 1.05 (0.62-1.78) R: 0.16 (0.05-0.52)	0.84 (0.55-1.28) L: 1.23 (0.75-2.01) R: 0.24 (0.08-0.74)	27/55	19/53

Ciardiello et al, 2016 [4] (CAPRI-GOIM)	Italy	Multicenter RCT	Jul 2009-Jun 2013	0-1	I:74 C:79	FOLFIRI + Cet	FOLFIRI + Cet	FOLFOX+ Cet	FOLFOX	N/A	N/A	0.81 (0.58–1.12)	0.86 (0.61–1.20)	N/A	N/A
Cremolini et al, 2016 [5] (MACBETH)	Italy	RCT	Nov 2011-Feb 2015	N/A	I:59 C:57	mFOLFOXIRI + Cet	mFOLFOXIRI + Cet	mFOLFOXIRI+ Cet	mFOLFOXIRI+B	N/A	N/A	0.69 (0.40-1.17)	N/A	40/59	43/57
Deng et al, 2015[6]	China	Retrospective single-center study	Jan 2009 -Apr 2013	N/A	I:8 C:18	5-FU (including capecitabine)/ oxaliplatin or irinotecan + B	5-FU (including capecitabine)/ oxaliplatin or irinotecan + Cet	5-FU (including capecitabine) / oxaliplatin or irinotecan + Cet	5-FU (including capecitabine) / oxaliplatin or irinotecan + B	N/A	N/A	N/A	N/A	6/8	12/18
Feng et al, 2016[7]	China	Retrospective single-center study	Jan 2012-Jan 2015	0-2	I:102 C:96 L: 30/31 R:33/26	mFOLFOX6 +Cet or FOLFIRI+ C	mFOLFOX6 + Cet or FOLFIRI+ Cet	FOLFIRI + Cet or mFOLFOX6 + Cet	FOLFIRI or mFOLFOX6	N/A	N/A	0.68 (0.49-0.93) L: 0.57 (0.31-1.06) R: 0.83 (0.48-1.41)	0.66 (0.45-0.97)	19/102	9/96
Hecht et al, 2015[8] (SPIRITT)	USA	Multicenter RCT	Nov 2006 - Dec 2010	0-1	I:91 C:91	Oxaliplatin-based + B	Oxaliplatin-based + B	FOLFIRI+ P	FOLFIRI+ B	N/A	N/A	1.01 (0.68-1.5)	1.06 (0.75-1.49)	28/87	16/83

Hsu et al, 2019 [9]	Taiwan	Retrospectively reviewed the medical records	July 2012-Dec 2016	0-1	I:46 C:56 L:89 R:13	FOLFIRI+ Cet	FOLFIRI+ B	FOLFIRI or mFOLFOX6	FOLFIRI or mFOLFOX6	FOLFIRI or IFL + B	FOLFIRI or IFL or irinotecan + Cet	0.43 (0.25-0.58)	0.54 (0.35-0.83) L: 0.6 (0.37-0.97) R: 0.24 (0.05-1.19)	10/46	13/56
Kubicka et al, 2013 [10] (ML18147)	Europe and Saudi Arabia	Multicenter RCT	Feb 2006-Jun 2010	0-2	I:151 C:165	Oxaliplatin-base + B or Irinotecan-base+ B	Oxaliplatin-base + B or Irinotecan-base + B	Irinotecan-base + B or Oxaliplatin-base + B	Irinotecan-base or Oxaliplatin-base	N/A	N/A	0.61 (0.49-0.77)	0.69 (0.53-0.9)	7/151	5/165
Modest et al, 2015[11] (FIRE-3)	Germany, Austria	Multicenter RCT	Jan 2007-Sep 2012	0-2	I:213 C:201 L: 170/139 R: 40/62	FOLFIRI+ Cet	FOLFIRI + B	FOLFOX+ B	Irinotecan + Cet	Third-line (later-line) treatment was defined as any anticancer drug that was not part of second-line (previous-line) therapy		0.68 (0.54-0.85) L: 0.65 (0.54-0.85) R: 1.02 (0.65-1.61)	0.70 (0.55-0.88) L: 0.65 (0.49-0.85) R: 1.02 (0.64-1.61)	42/213	42/201
Passardi et al, 2017 [12] (ITACa)	Italy	Multicenter RCT	Nov 2007-Mar 2012	0-2	I:23 C:25	FOLFIRI + B or FOLFOX4+ B	FOLFIRI + B or FOLFOX4+ B	FOLFOX4+ Cet or FOLFIRI + Cet	FOLFOX4 or FOLFIRI	N/A	N/A	0.64 (0.35-1.16)	1.30 (0.70–2.44)	7/23	4/25
Peeters et al, 2013[13] (PRIME)	USA, UK, Europe	Multicenter RCT	N/A	N/A	N/A	FOLFOX4 +P	FOLFOX4 +P	Anti-VEGFR + FOLFOX4	FOLFOX4	N/A	N/A	N/A	0.64 (0.44–0.94)	N/A	N/A

Peeters et al, 2017 [14] (PEAK, PRIME, 181 study)	USA, UK, Europe	Prospective–retrospective analysis of a randomized, multicenter phase III study	Apr 2009 - Dec 2011 Aug 2006 - Aug 2009 Jun 2006- Mar 2008	0-2	I:66 C:38	PEAK: mFOLFOX6 + P PRIME: FOLFOX4 + P	PEAK: mFOLFOX6 + B 181 study: oxaliplatin + B	PEAK: chemo+ VEGFI PRIME: chemo + VEGFI	PEAK: chemo + EGFRI 181 study: FOLFIRI + P	N/A	N/A	N/A	0.65 (0.42 - 1.03)	N/A	N/A
Peeters et al, 2014[15] (20050181 study)	Europe	Multicenter RCT	June 2006- Mar 2008	0-2	I:297 C:286	Oxaliplatin-base + B	Oxaliplatin-base + B	FOLFIRI+ P	FOLFIRI	N/A	N/A	0.65 (0.45-0.96)	0.71 (0.48-1.05)	107/297	28/286
Rosati et al, 2015 [16]	Italy	Multicenter RCT	Dec 2009- Jul 2015	N/A	I: 54 C:54	FOLFIRI+ B	FOLFIRI+ B	Irinotecan + Cet	FOLFOX-4	FOLFOX-4	Irinotecan + Cet	0.85 (0.56-1.28)	0.79 (0.52-1.22)	21/52	15/52
Shitara et al, 2016[17] (WJOG 6210G)	Japan	Multicenter RCT	Apr 2011- Feb 2014	0-1	I:58 C:59	Oxaliplatin-base + B	Oxaliplatin-base + B	FOLFIRI+ B	FOLFIRI+ P	N/A	N/A	1.14 (0.78-1.66)	1.16 (0.76-1.77)	3/53	24/52

Abbreviations: Chemo=chemotherapy; B= bevacizumab; Cet=cetuximab; C: Comparison; 5-FU= fluorouracil; FOLFOX = oxaliplatin, leucovorin and fluorouracil; FOLFIRI= irinotecan, leucovorin and fluorouracil; FOLFOXIRI= irinotecan, oxaliplatin, leucovorin, fluorouracil; I: Intervention; mFOLFOX=modified FOLFOX; mFOLFOXIRI=modified FOLFOXIRI; n= event number; N=total number ; N/A= not applicable; ORR= Objective response rate; P=Panitumumab; VEGFI: vascular endothelial growth factor receptor inhibitors; EGFRI: epidermal growth factor receptor inhibitor

1. *Cetuximab: new drug. Metastatic colorectal cancer: an inappropriate evaluation.* Prescrire Int, 2005. **14**(80): p. 215-7.
2. Buchler, T., et al., *Sequential therapy with bevacizumab and EGFR inhibitors for metastatic colorectal carcinoma: a national registry-based analysis.* Cancer Manag Res, 2019. **11**: p. 359-368.
3. Cascinu, S., et al., *Treatment sequence with either irinotecan/cetuximab followed by FOLFOX-4 or the reverse strategy in metastatic colorectal cancer patients progressing after first-line FOLFIRI/bevacizumab: An Italian Group for the Study of Gastrointestinal Cancer phase III, randomised trial comparing two sequences of therapy in colorectal metastatic patients.* Eur J Cancer, 2017. **83**: p. 106-115.
4. Ciardiello, F., et al., *Cetuximab continuation after first progression in metastatic colorectal cancer (CAPRI-GOIM): a randomized phase II trial of FOLFOX plus cetuximab versus FOLFOX.* Ann Oncol, 2016. **27**(6): p. 1055-61.
5. Cremolini, C., et al. *Modified FOLFOXIRI (mFOLFOXIRI) plus cetuximab (cet), followed by cet or bevacizumab (bev) maintenance, in RAS/ BRAF wt metastatic colorectal cancer (mCRC): the phase II randomized MACBETH trial by GONO*. Annals of oncology, 2016. **27**, DOI: 10.1093/annonc/mdw370.11.
6. Deng, Y., et al., *Survival of patients with KRAS wild-type metastatic colorectal cancer is identical after sequential treatment with cetuximab and bevacizumab regardless of the sequence - A retrospective single-center study.* Gastroenterol Rep (Oxf), 2015. **3**(4): p. 339-43.
7. Feng, Q., et al., *Efficacy of continued cetuximab for unresectable metastatic colorectal cancer after disease progression during first-line cetuximab-based chemotherapy: a retrospective cohort study.* Oncotarget, 2016. **7**(10): p. 11380-96.
8. Hecht, J.R., et al., *SPIRITT: A Randomized, Multicenter, Phase II Study of Panitumumab with FOLFIRI and Bevacizumab with FOLFIRI as Second-Line Treatment in Patients with Unresectable Wild Type KRAS Metastatic Colorectal Cancer.* Clin Colorectal Cancer, 2015. **14**(2): p. 72-80.
9. Hsu, H.C., et al., *Sequential cetuximab/bevacizumab therapy is associated with improved outcomes in patients with wild-type KRAS exon 2 metastatic colorectal cancer.* Cancer Med, 2019. **8**(7): p. 3437-3446.
10. Kubicka, S., et al., *Bevacizumab plus chemotherapy continued beyond first progression in patients with metastatic colorectal cancer previously treated with bevacizumab plus chemotherapy: ML18147 study KRAS subgroup findings.* Ann Oncol, 2013. **24**(9): p. 2342-9.
11. Modest, D.P., et al., *Impact of Subsequent Therapies on Outcome of the FIRE-3/AIO KRK0306 Trial: First-Line Therapy With FOLFIRI Plus Cetuximab or Bevacizumab in Patients With KRAS Wild-Type Tumors in Metastatic Colorectal Cancer.* J Clin Oncol, 2015. **33**(32): p. 3718-26.
12. Passardi, A., et al., *Impact of second-line cetuximab-containing therapy in patients with KRAS wild-type metastatic colorectal cancer: results from the ITACa randomized clinical trial.* Sci Rep, 2017. **7**(1): p. 10426.
13. Peeters, M., et al. *Impact of post-progression anti-vascular endothelial growth factor-containing therapy on survival in patients with metastatic colorectal cancer: data from the PRIME study*. European journal of cancer., 2013. **49**, S18 DOI: 10.1016/S0959-8049%2813%2970139-4.
14. Peeters, M., et al., *Exploratory pooled analysis evaluating the effect of sequence of biological therapies on overall survival in patients with RAS wild-type metastatic colorectal carcinoma.* ESMO Open, 2018. **3**(2): p. e000297.
15. Peeters, M., et al., *Final results from a randomized phase 3 study of FOLFIRI ± panitumumab for second-line treatment of metastatic colorectal cancer.* Annals of Oncology, 2014. **25**(1): p. 107-116.
16. Rosati, G., et al. *A phase III multicenter trial comparing two different sequences of second/third line therapy (irinotecan/cetuximab followed by FOLFOX-4 vs. FOLFOX-4 followed by irinotecan/cetuximab in K-RAS wt metastatic colorectal cancer (mCC) patients refractory to FOLFIRI/Bevacizumab*. Annals of oncology., 2015. **26**, vi2 DOI: 10.1093/annonc/mdv335.3.
17. Shitara, K., et al., *Randomized study of FOLFIRI plus either panitumumab or bevacizumab for wild-type KRAS colorectal cancer-WJOG 6210G.* Cancer Sci, 2016. **107**(12): p. 1843-1850.

Reference:

Supplementary Table S3 ROBINS-I for the assessment of the quality of the included observational studies

**Study**	Buchler *et al.*2019 (1)	Deng *et al.*2015 (2)	Feng *et al.*2016 (3)	Hsu *et al.*2019 (4)
**Bias due to confounding**	Moderate	Moderate	Moderate	Moderate
**Bias in selection of participants into the study**	Moderate	Moderate	Moderate	Moderate
**Bias in classification of interventisons**	Moderate	Moderate	Moderate	Moderate
**Bias due to deviations from intended intervention**	Low	Low	Low	Low
**Bias due to missing data**	Low	No information	Low	Moderate
**Bias in measurement of outcomes**	Moderate	Moderate	Moderate	Moderate
**Bias in selection of the reported result**	Low	Low	Low	Low
**Overall bias**	Moderate	Moderate	Moderate	Moderate

1. Buchler T, Chloupkova R, Poprach A, Fiala O, Kiss I, Kopeckova K, Dusek L, Veskrnova V, Slavicek L, Kohoutek M, *et al:* Sequential therapy with bevacizumab and EGFR inhibitors for metastatic colorectal carcinoma: a national registry-based analysis. Cancer Manag Res. 11: 359-368, 2019.
2. Deng Y, Cai Y, Lin J, Jiang L and Hu H: Survival of patients with KRAS wild-type metastatic colorectal cancer is identical after sequential treatment with cetuximab and bevacizumab regardless of the sequence - A retrospective single-center study. Gastroenterol Rep (Oxf) 3: 339-343, 2015.
3. Feng Q, Wei Y, Ren L, Zheng P, Yu Y, Ye Q, Ding J, Chen J, Chang W, Zhong Y, *et al:* Efficacy of continued cetuximab for unresectable metastatic colorectal cancer after disease progression during first-line cetuximab-based chemotherapy: A retrospective cohort study. Oncotarget 7: 11380-11396, 2016.
4. Hsu HC, Liu YC, Wang CW, Chou WC, Hsu YJ, Chiang JM, Lin YC and Yang TS: Sequential cetuximab/bevacizumab therapy is associated with improved outcomes in patients with wild-type KRAS exon 2 metastatic colorectal cancer. Cancer Med 8: 3437-3446, 2019.
